# Altered Proteins in the Hippocampus of Patients with Mesial Temporal Lobe Epilepsy

**DOI:** 10.3390/ph11040095

**Published:** 2018-09-30

**Authors:** Daniele Suzete Persike, Jose Eduardo Marques-Carneiro, Mariana Leão de Lima Stein, Elza Marcia Targas Yacubian, Ricardo Centeno, Mauro Canzian, Maria José da Silva Fernandes

**Affiliations:** 1 Departamento de Neurologia/Neurocirurgia, Escola Paulista de Medicina, Universidade Federal de São Paulo–UNIFESP, Rua Pedro de Toledo, 669, CEP, São Paulo 04039-032, Brazil; daniele_persike@protonmail.com (D.S.P.); edumarques83@gmail.com (J.E.M.-C.); yacubian@terra.com.br (E.M.T.Y.); ricardoscenteno@gmail.com (R.C.); 2 Department of Medicinal Chemistry, College of Pharmacy, University of Dohuk-UoD, Kurdistan Region 1006AJ, Iraq; 3 INSERM U1114, Neuropsychologie Cognitive et Physiopathologie de la Schizophrenie, 1 pl de l’Hopital, 67091 Strasbourg, France; 4 Departamento de Micro-Imuno-Parasito, Disciplina de Biologia Celular, Escola Paulista de Medicina, UNIFESP, São Paulo 04039-032, Brasil; mikota_bio@yahoo.com.br; 5 Instituto do Coração (INCOR), Departamento de Anatomia Patológica, Faculdade de Medicina da Universidade de São Paulo (FMUSP), São Paulo 04039-032, Brasil; mauroczn@hotmail.com

**Keywords:** temporal lobe epilepsy, hippocampus, proteomics, biomarkers, comorbidities

## Abstract

Mesial temporal lobe epilepsy (MTLE) is usually associated with drug-resistant seizures and cognitive deficits. Efforts have been made to improve the understanding of the pathophysiology of MTLE for new therapies. In this study, we used proteomics to determine the differential expression of proteins in the hippocampus of patients with MTLE compared to control samples. By using the two-dimensional electrophoresis method (2-DE), the proteins were separated into spots and analyzed by LC-MS/MS. Spots that had different densitometric values for patients and controls were selected for the study. The following proteins were found to be up-regulated in patients: isoform 1 of serum albumin (ALB), proton ATPase catalytic subunit A (ATP6V1A), heat shock protein 70 (HSP70), dihydropyrimidinase-related protein 2 (DPYSL2), isoform 1 of myelin basic protein (MBP), and dihydrolipoamide S-acethyltransferase (DLAT). The protein isoform 3 of the spectrin alpha chain (SPTAN1) was down-regulated while glutathione S-transferase P (GSTP1) and protein DJ-1 (PARK7) were found only in the hippocampus of patients with MTLE. Interactome analysis of the nine proteins of interest revealed interactions with 20 other proteins, most of them involved with metabolic processes (37%), presenting catalytic activity (37%) and working as hydrolyses (25%), among others. Our results provide evidence supporting a direct link between synaptic plasticity, metabolic disturbance, oxidative stress with mitochondrial damage, the disruption of the blood–brain barrier and changes in CNS structural proteins with cell death and epileptogenesis in MTLE. Besides this, the presence of markers of cell survival indicated a compensatory mechanism. The over-expression of GSTP1 in MTLE could be related to drug-resistance.

## 1. Introduction

Epilepsy is a neurological condition affecting more than 50 million people, with a possible 2.4 million new cases per year. Up to 10% of individuals globally have had a seizure during their lives [1]. Temporal lobe epilepsy (TLE) is commonly sub-classified into mesial temporal lobe epilepsy (MTLE) and lateral temporal neocortical epilepsy, accounting for about 40% of all cases of epilepsy [2]. 

Hippocampal sclerosis (HS) is the main neuropathological abnormality associated with MTLE, although abnormalities in the thalamus and neocortex are also frequent in these patients [3,4]. Abnormalities in the amygdala have been reported in patients with MTLE who frequently present psychiatric comorbidities such as depression, anxiety and schizophrenia [5,6]. 

In 30% of MTLE cases, seizures are resistant to antiepileptic drugs and rates of neurobehavioral comorbidity are higher in these patients than in the general population [7]. Psychiatric symptoms such as depression, psychosis, hyperactivity and anxiety are common in MTLE, but the mechanisms involved in these comorbidities are unknown [8]. Other comorbidities in epilepsy include cognitive dysfunction, sleep disturbance, dementia, vascular pathology and stroke. Patients under these conditions have poor prognosis, a poor response to antiepileptic drugs and a poor quality of life [9]. Thus, there is a growing search for biomarkers to allow more precise and earlier diagnoses, as well as improved prevention and treatment.

Proteomics has been an essential tool for studying changes in the protein profiles of signaling pathways in normal and pathological conditions. It has been widely used in clinical and preclinical research with the purpose of providing comprehensive knowledge about molecular pattern alterations and as a guide in the identification of biomarkers involved with epileptogenesis. A growing number of studies analyzing samples of patients with MTLE (brain tissue, blood or cerebrospinal fluid) or of experimental models of MTLE have identified alterations in proteins related to neurotransmitters, cellular signaling, and glucose metabolism [10,11,12,13,14,15,16]. In the present study, we performed a broad analysis of the data, partially presented as a short communication [17] referring to human hippocampal proteomic analysis obtained from patients with MTLE and depression as a comorbidity in 50% of cases, compared to control samples obtained from autopsy. Key proteins related to the mechanisms of cell damage caused by MTLE, as well as compensatory mechanisms of cell damage, were identified. Some of the identified proteins are also involved with mechanisms such as drug-resistance and comorbidity in patients with MTLE.

## 2. Results

### 2.1. Comparative Proteomic Analysis

**Figure 1:**
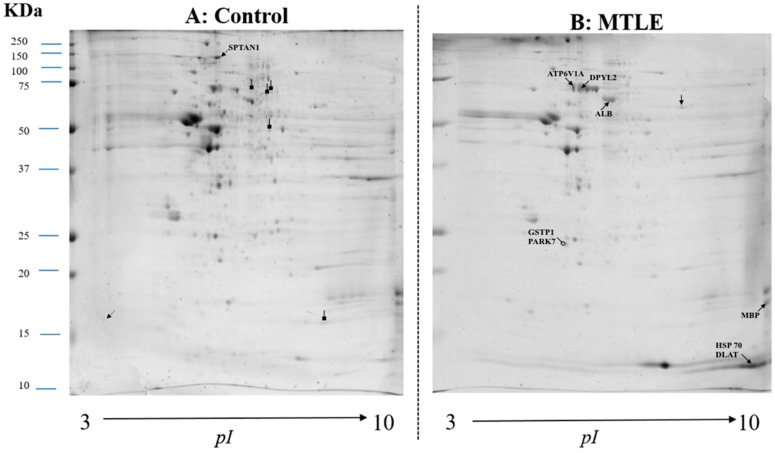
A representative 2D-PAGE image showing all 16 spots of protein of interest from control (*n* = 10) (**A**) and patients with mesial temporal lobe epilepsy (MTLE) (*n* = 6) (**B**). (**A**) Arrows with black square tips are the proteins detected only in the control group (5) which were not satisfactorily identified. Up-regulated proteins are shown as dotted arrows (2), one of which was identified as the spectrin alpha chain (SPTAN1). (**B**) Arrows with empty square tips are the proteins detected only in MTLE patients (2) identified as glutathione S-transferase P (GSTP1) and protein DJ-1 (PARK7). Up-regulated proteins are shown as filled arrows (7) identified as proton ATPase catalytic subunit A (ATP6V1A), heat shock protein 70 (HSP70), dihydropyrimidinase-related protein 2 (DPYSL2), isoform 1 of myelin basic protein (MBP) isoform 1 of serum albumin (ALB), heat shock protein 70 (HSP70), and dihydrolipoamide S-acethyltransferase (DLAT).

Using a 2-DE based proteomic approach, we determined the protein expression profiles in the hippocampus of patients with MTLE compared to the control hippocampi removed from autopsies. A total of 40 two-dimensional electrophoresis gels corresponding to 10 samples per group, in duplicate, were analyzed simultaneously and matched in the same set. The gels were analyzed separately by study group for number, location and densitometry of the spots, and similar gels were chosen for the study (6 for MTLE and 10 for control, both in duplicate). In general, we detected approximately 192–269 spots in each gel. The average number of spots detected in the 2-DE gels was 215 ± 21 in patients and 237 ± 32 in control samples. The comparative analysis of 2-DE gels showed 16 spots differentially expressed between groups. Figure 1 shows a representative 2-DE gel image of control samples and of epileptic hippocampal samples. In the 2-DE images of epileptic groups, seven more spots were up-regulated compared to the control, of which six were satisfactorily identified as heat shock protein 70 (HSP70), proton ATPase catalytic subunit A (ATP6V1A), dihydropyrimidinase-related protein 2 (DPYSL2), myelin basic protein (MBP), isoform 1 of serum albumin (ALB), and dihydrolipoamide S-acethyltransferase (DLAT). Two spots were present only in epileptic condition, identified as glutathione S-transferase P (GSTP1) and protein DJ-1 (PARK7). In gels of the control group, two spots were up-regulated compared to epileptic samples, and one of them was identified as spectrin alpha chain (SPTAN1). The second spot was not identified satisfactorily, as well as the five spots which were found only in control (≥2-fold change, *t*-test, *p* < 0.05). As shown in Table 1, nine distinct proteins identified (IP) in 2-DE gels of patients using ESI-LC and MS/MS analysis were differentially expressed compared to the control group, corresponding to five unique and well resolved proteins and four overlapping proteins.

### 2.2. Interactome

**Figure 2:**
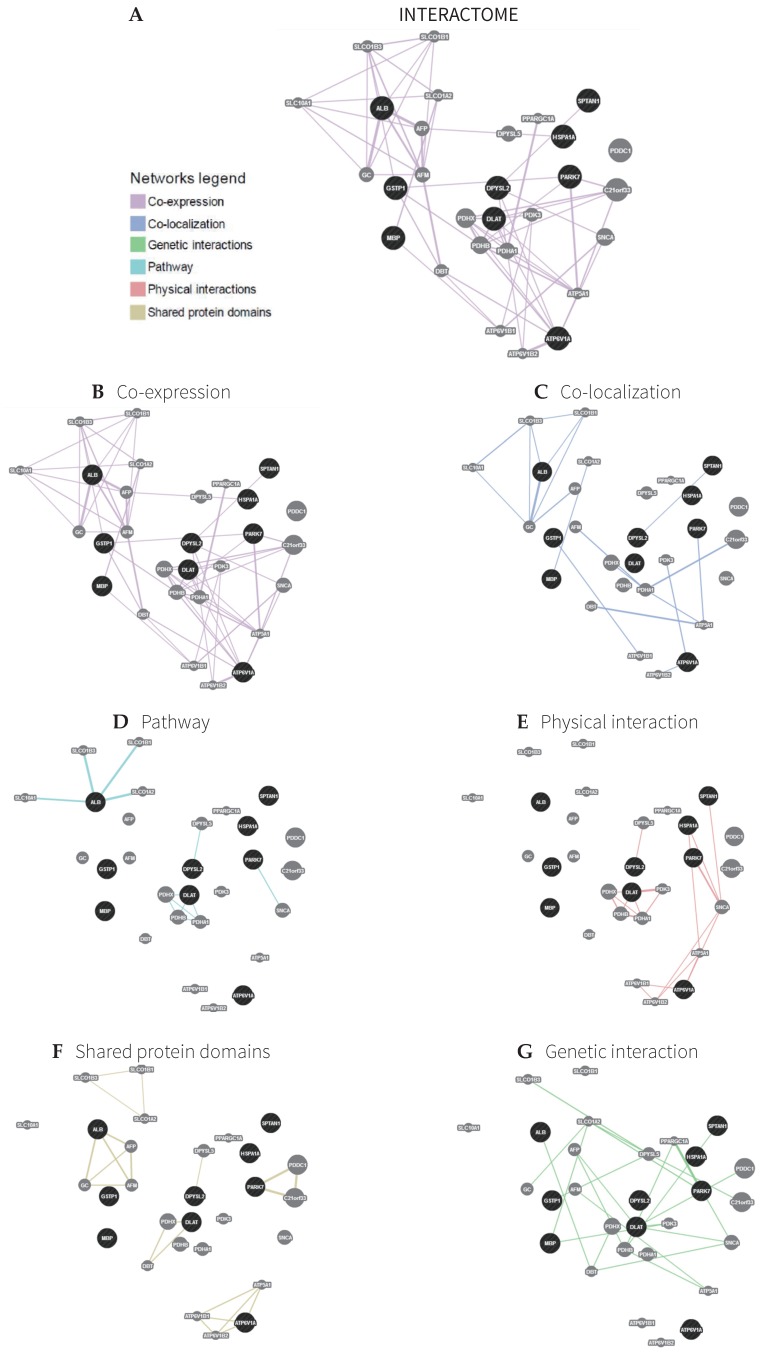
Schematic representation of the interactome showing the network between our proteins of interest with 20 other proteins. Proteins of interest are indicated in black and interacting proteins are indicated in gray. The size of the gray spots indicates the weight of the interactions.

**Figure 3:**
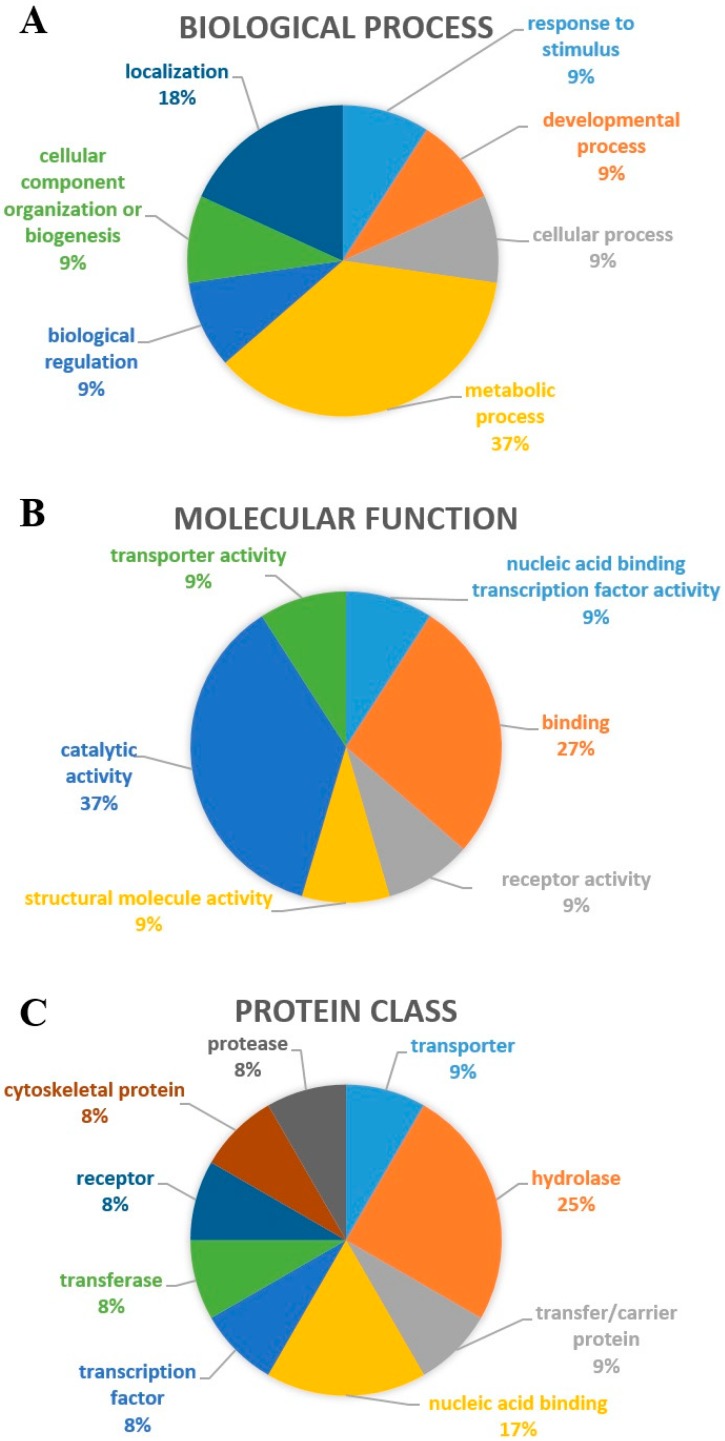
Schematic profile indicating the proportion (%) of the proteins of interest in each category: biological process (**A**); molecular function (**B**); and protein class (**C**).

Through the GENEmania software, we analyzed the nine proteins of interest and found seven biological processes and six different molecular functions associated to them (GSTP1, DPYSL2, ATP6V1A, PARK7, DLAT, ALB, SPTAN1, MBP, and HSP70). These proteins interact with 20 other proteins: Parkinson disease 7 domain containing 1 (PDDC1), chromosome 21 open reading frame 33 (C21orf33), pyruvate dehydrogenase complex component X (PDHX), synuclein alpha (SNCA), pyruvate dehydrogenase beta (PDHB), pyruvate dehydrogenase alpha 1 (PDHA1), dihydropyrimidinase-like 5 (DPYSL5), alpha-fetoprotein (AFP), afamin (AFM), group-specific component vitamin D binding protein (GC), pyruvate dehydrogenase kinase 3 (PDK3), dihydrolipoamide branched chain transcyclase E2 (DBT), solute carrier organic anion transporter family 1B3 (SLCO1B3), solute carrier organic anion transporter family 1A2 (SLCO1A2), solute carrier organic anion transporter family 1B1 (SLCO1B1), ATPase H+ transporting lysosomal 56/58 kDa V1 subunit B1 (ATP6V1B1), ATPsynthase H+ transporting mitochondrial F1 complex alpha subunit (ATP6A1), ATPase H+ transporting lysosomal 56/58 kDa V1 subunit B2 (ATP6V1B2), solute carrier family 10 (SLC10A1), and peroxisome proliferator-activated receptor gamma coactivator 1 alpha (PPARGC1A). Table 2 shows the functions of the proteins of interest and Table 3 shows the function of proteins with which the proteins of interest make networks. The data also show the number of genes in the network and the total number of genes (from the genome) involved with each function. A computational network interaction between the proteins of interest with the 20 other identified proteins is shown in Figure 2A–G, distinguishing interactions based on co-expression, co-localization, pathways, physical interaction shared proteins, and genetic interactions. Figure 3 shows the distribution of target proteins according to their: biological process (Figure 3A), molecular function (Figure 3B) and protein class (Figure 3C). Regarding the biological process, 37% are involved with metabolic processes, followed by localization processes (18%), cellular processes (9%), biological regulation (9%), developmental processes (9%), response to stimuli (9%), and organization of cellular components (9%) (Figure 3A). The interactome analysis also shows that target proteins are mainly related to catalytic activity (37%), followed by a binding function (27%), transport function (9%), structural molecular activity (9%), nucleic acid binding transcription factor function (9%), and receptor activity function (9%) (Figure 3B). Finally, the class distribution shows that most are hydrolases (25%), followed by nucleic acid ligands (17%), cytoskeletal proteins (8%), receptors (8%), proteases (8%), transporters (9%), carriers and transferases (9%), and transcription factors (8%) (Figure 3C).

### 2.3. Western Blot Validation

**Figure 4:**
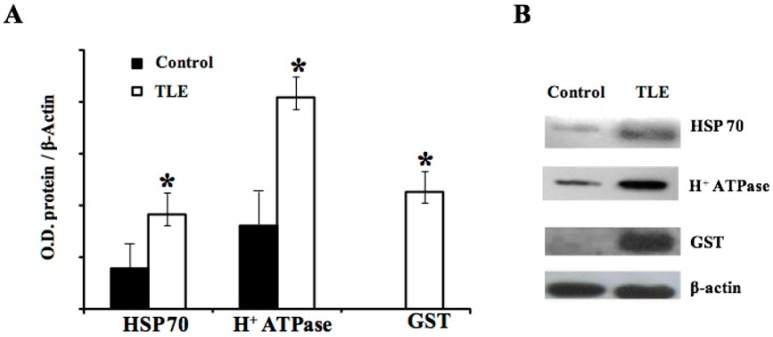
Western blot analysis to validate the differential displays for HSP70, H^+^-ATPase, and GST in MTLE and control samples. β-actin was used as an endogenous control for protein expression. Densitometry analysis was performed using Densirag software. (**A**) Band densities of samples (control, *n* = 4; and MTLE, *n* = 5) were digitized to measure optical density (mean ± SD). (**B**) A representative Western blot of HSP70, H^+^-ATPase, and GST expression.

Western blot testing was used to validate the data of the proteomic analysis and to make the predictive results more reliable. To accomplish this, a Western blot was made using monoclonal and polyclonal antibodies against three proteins differentially expressed in the epileptic tissue compared to the control: HSP 70, H^+^-ATPase, and GST. As shown in Figure 4, a significant increase of HSP 70 (*p* < 0.01) and H^+^-ATPase (*p* < 0.05) was found in the MTLE group compared to the control group, and the expression of GST was observed only in the MTLE group.

## 3. Discussion

The present study shows that nine proteins are differentially expressed in the hippocampi of patients with MTLE compared to the control samples. Proteins that were up-regulated were DPYSL2, ATP6V1A, DLAT, ALB, MBP, and HSP70. The levels of SPTAN1 were significantly decreased in the hippocampi of patients with MTLE compared to the control, while GSTP1 and PARK7 were detected only in epileptic tissue. Most of these proteins do not have a well-defined role in the epileptic process. Epilepsy is often associated with defects in ion channel proteins [31]. The fact that we did not find alterations in ion channel proteins may be explained by specific features of MTLE or by the insufficient amounts of samples that we have analyzed.

### 3.1. Neuronal Development and Plasticity

DPYSL2 was found to be up-regulated in all patients with MTLE compared to the control. Previous studies also reported an up-regulation of DPYSL2 in autopsy and human biopsy tissue from epileptic patients [15]. This protein was also up-regulated in the MTLE model induced by pilocarpine compared to the control, and treatment with a disease modifier neuromodulator, carisbamate, normalized the expression of DPYSL2 [32]. Carisbamate treatment reversed the increase in the protein expression and caused a change in the expression of limbic seizures for absence seizures in treated animals [32]. DPYSL2, a member of the phosphoproteins found in the cytosol, participates in the process involved with neuronal migration, the development of neuronal polarity, axon growth and guidance [33]. DPYSL2 gene has also been associated with susceptibility to psychiatric disorders, such as schizophrenia [34], or with psychiatric comorbidity, as shown in Alzheimer’s disease [22]. Contrary to what is observed in the brains of patients with schizophrenia or Alzheimer’s disease, DPY was up-regulated in all hippocampal samples of patients with MTLE, including those who experienced depression as a psychiatric comorbidity (50% of total samples). Increased expression of this protein in the hippocampi of patients with MTLE may indicate the presence of neurogenesis and synaptic plasticity, currently observed in these patients as discussed by Curia et al. [35]. Considering its role in axonal growth and guidance, we can hypothesize that it can be involved in the process of neuronal sprouting and seizures generation.

The isoform 3 of spectrin (SPTAN1), a protein involved with synaptic plasticity, was down-regulated in MTLE compared to the control sample. It has been shown that one of the functions of SPTAN1 is to anchor the NMDA receptor to the membrane. Changes in the expression of spectrin have been associated with abnormal LTP and cognition [20]. It is known that spectrin, as well as DPYSL2, is involved in the growth of axons and neurites, as well as in synaptic plasticity [23]. The present data suggest that the down-regulation of spectrin can disturb glutamatergic transmission by changing the anchoring of the NMDA receptors to the membrane.

Studies have shown that seizures can induce neurogenesis in the dentate gyrus, and newborn neurons can integrate with the hippocampal network and increase the susceptibility to seizures in rats [36,37]. Besides that, increased expression of plasticity-associated proteins, growth-associated protein-43 (GAP-43), microtubule-associated protein 1B (MAP1B), and tissue plasminogen activator (tPA) mRNAs was also shown in hippocampus of animals in conditions of increased seizure susceptibility [38]. Despite the evidence of neurogenesis in the hippocampus of experimental models of epilepsy, in patients with MTLE, neurogenesis is detected only in the early stages of the disease. Perhaps this is the reason no markers of neurogenesis were detected in the present study. The literature is still very controversial about the presence of neurogenesis in the sub-ventricular zone (SVZ) or in the sub-granular zone (SGZ) of hippocampal formation of patients with MLE studied in later stages of the disease, when the cognitive deficit is already present [39].

### 3.2. Neuronal Excitability

HSP70, a stress-inducible chaperone, is increased in patients with MTLE and this change can either represent a compensatory mechanism or simply reflect an increase in the protein synthesis as a consequence of new protein folding [21]. Increased expression of HSP70 has been reported by other authors in brain samples of patients with intractable epilepsy, and in experimental models of epilepsy [40,41]. Recent study has shown that heat shock cognate 71 kDa protein (Hspa8) is up-regulated in the hippocampus of the lithium–pilocarpine model of temporal lobe epilepsy [32].

The vacuolar H^+^-ATPase was also found increased in the hippocampi of patients with MTLE compared to the control. H^+^-ATPase is an evolutionarily ancient enzyme involved in many biochemical mechanisms such as neurotransmitter release [42], the regulation of neurotransmitter storage in response to oxidative stress [43], the acidification of synaptic vesicles after exocytosis [44], and the active transport of metabolites [42]. The up-regulation of H^+^-ATPase can reflect an increase in the dynamics of synthesis, storage and release of neurotransmitters present in epileptic tissue and therefore increased excitation. Similar changes were found in preclinical conditions using the TLE model in rats [32].

Glutathione S-transferase P (GSTP1), and Parkinson protein 7 (PARK-7) were exclusively detected in the hippocampi of patients with MTLE, not in control samples. This is notable because GSTP1 and PARK-7 play an important antioxidant role following brain injury [22,25,45]. GSTP1 has been associated with the inactivation of antiepileptic drugs in the liver [22]. Besides this, this protein belongs to a family of enzymes that has an important role in detoxification, whose mechanism depends on glutathione. In line with our results, Shang et al. [23] showed an up-regulation of GSTP1 in the brain of patients with drug-resistant seizures, suggesting that this enzyme may be responsible for the poor penetration of antiepileptic drugs into the brain and, therefore, represents a possible mechanism of intractability in seizures. Because of this, the protein may represent an important target to study drug-resistance associated with MTLE.

Antioxidative stress is one of the multi-protective functions of PARK-7 [24,25]. Previous study has shown increases in the expression of PARK-7 and HSP70 in transgenic mice to express Parkinson’s disease related to neuroprotection caused by the practice of physical exercise [46]. Thus, the up-regulation of PARK-7 and HSP70 found in MTLE patients indicates the presence of neuroprotective mechanisms in response to MTLE.

### 3.3. Mitochondria and Bioenergetics

In addition to the proteins described above, proteins involved with neuronal metabolism and energy production can also have a role in neuronal excitability. DLAT, which is part of the pyruvate dehydrogenase complex (PDHc), is up-regulated in patients with MTLE. Besides this, the interactome points to a modulation by DLAT of PDHX, PDH1 and PDK3.

The reactions of the PDHc serve to interconnect the metabolic pathways of glycolysis, gluconeogenesis and fatty acid oxidation to the Krebs cycle. The PDHc is comprised by three distinct enzyme activities: PDH, DLAT, and dihydrolipoamide dehydrogenase (DLD). The active state of the PDH is regulated by the phosphorylation process, being most active in the dephosphorylated state. Four PDH kinases are responsible for the phosphorylation of PDHc. Under high cellular energy, when ATP, NADH and acetyl-CoA are increased, the activity of these kinases enhances [26]. As a result, aerobic tissues such as in the brain are most sensitive to alterations in components of the PDHc, since the energetic metabolism of this organ is dependent on a normal conversion of pyruvate to acetyl-CoA.

Several proteins involved in the glycolytic via were also found to be up-regulated in temporal lobe epilepsy model induced by lithium–pilocarpine [32]. It is well known that ATP demand and production is critical in epilepsy, because epileptiform activity induces large ionic conductance and depletes vesicular stores, and the restoration of these changes, i.e., the restoration of cellular homeostasis, is an energy-demanding process [47]. In addition, epilepsy has been associated with metabolic and homeostatic changes leading to energy failure and neuronal damage [47]. Inhibition of the glycolytic pathway by means of a diet that induces ketogenesis has been an effective strategy for controlling refractory seizures [48,49]. Peptides such as insulin, leptin, ghrelin, and adiponectin are released through food intake or fasting. Insulin has been associated with seizure facilitation, while leptin, ghrelin, and adiponectin have been related to seizure suppression [49].

**Figure 5:**
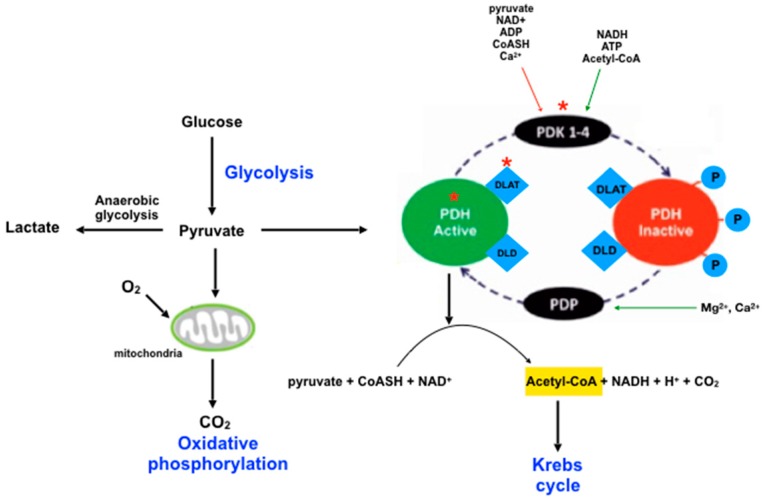
Schematic representation of the main altered metabolic pathways in MTLE revealed by proteomic analysis: glycolysis, pyruvate metabolism, Krebs cycle and oxidative phosphorylation. The affected proteins pyruvate dehydrogenase (PDH), PDH kinases (PDK) and dihydrolipoamide S-acetyltransferase (DLAT) are indicated by red asterisk.

As shown in Table 4, several proteins from glycolysis, the Krebs cycle, and oxidative phosphorylation can be modulated by the action of DLAT in patients with MTLE. A schematic representation showing the main alterations in the metabolic pathways of patients with MTLE obtained in the present study is shown in Figure 5.

### 3.4. Integrity of the Blood–Brain Barrier and Myelination

Additionally, the increased level of isoform 1 of myelin basic protein (MBP) and isoform 1 of albumin (ALB) in MTLE are indicative of disturbances in the myelination mechanism and a disruption in the permeability of the blood–brain barrier (BBB). MBP is the main component of the myelin sheath of oligodendrocytes in the central nervous system, making up 1/3 of total proteins. Proteins associated with myelin, including MBP, are decreased in the epileptic focus of patients with intractable epilepsy, suggesting that the alteration can disturb the transmission of nerve impulse [19]. Contrary to the study by He et al. [15], an increase of MBP was observed in the present work, suggesting the entry of this protein in the CNS due to the breakdown in the BBB. Similar results reported in animal models of epilepsy also showed increased expression of MBP in the hippocampus, indicating a breakdown in the BBB [18,50]. The presence of ALB in the hippocampus of patients with MTLE and in experimental models of epilepsy are also evidence of disruption of the BBB. The presence of albumin following disruption of the BBB by seizures has been shown by several authors [19,20,51]. Previous report has shown that neuronal death and ischemic-like lesion that occur in the hippocampus following SE can be prevented by the specific inhibition of matrix metalloproteinase-12, a protein supposed to be involved in BBB leakage [52].

### 3.5. Interactome

In the present study, an interactome analysis pointed to 20 proteins interacting with the nine proteins of interest (GSTP1, DPYSL2, ATP6V1A, PARK7, DLAT, ALB, SPTAN1, MBP, and HSP70). The data obtained in the interaction study between the proteins of interest with the 20 proteins found by the analysis reinforce the results obtained through the proteomic study. Among the findings, the following should be highlighted, due to their known functional relationship, as previously discussed: GSTP1 and ATP6V; PARK-7 and SNCA; DYSL2 and HSPA1A; DLAT and PDHc (A1, B, X); and PDK.

By analyzing the functional annotation of the 20 found proteins, 37% of the proteins are involved with metabolic processes (Figure 3A), wherein most of them (37%) are related to catalytic activity, while 27% are involved in the binding process (Figure 3B). Furthermore, according to protein class, 25% of these proteins are hydrolases, and 17% are nucleic acid binding proteins (Figure 3C). 

Taken together, our results corroborate with recent data in which authors used rat models to study epileptogenesis by means of proteomic analysis [51]. The authors showed an increase in the level of protein involved with cell stress and death. Extracellular matrix remodeling was also evident in the late phase of the epileptic process [51].

The physiology of the cell can be understood as the result of thousands of proteins acting together to orchestrate the cellular response. Protein communities including disease networks can be revealed by the interactome. Knowing the proteomic architecture of the human hippocampus is important because it expands the knowledge about mechanisms that are disturbed by disease conditions and contributes to the process of identifying molecular targets for new therapies.

However, we emphasize that studies employing human tissue are limited by the relatively small amounts of tissue obtained during surgical procedures as well as bioethical concerns.

## 4. Materials and Methods

### 4.1. Human Tissue

All experiments had the approval of the institutional ethics committee (CEP-UNIFESP, 1692/05). Surgical specimens from patients with intractable epilepsy were subjected to standard cortico-amygdalo-hippocampectomy at the Surgical Center of São Paulo Hospital (UNIPETE/HSP-HU, Brazil). All cases showing neoplasm, vascular malformations, post-traumatic and ischemic lesions on preoperative MRI were discarded from the study. The selected patients (*n* = 6) had detailed anamnesis, video-EEG recordings and MRI studies. Following these procedures, the epileptogenic zone was delineated by neurosurgeons. As shown in Table 4, the age of the patients with TLE ranged from 30 to 56 years (42.2 ± 9.9 years) with 33.3% female cases and 66.6% male cases.

Hippocampal samples of the control group were obtained from autopsied patients (<6 h from postmortem), and were devoid of psychiatric diseases and changes in the central nervous system based on clinical history or under routine histological examination by experienced pathologist. Of the sampled autopsy tissue (40% female, 60% male), the age of the subjects at death ranged from 28 to 86 (56 ± 18; *n* = 10). Demographic and clinical data of control are shown in Table 5.

A pathologist specially trained by a neurosurgeon to carry out the removal of hippocampal formation performed the autopsies. Thus, similar hippocampal samples from epileptic patients and autopsied subjects were studied. All patients and families had signed a consent form authorizing the utilization of the tissues.

### 4.2. Sample Preparation

About 100 mg (wet weight) of hippocampal tissue was homogenized for protein extraction using a buffer (5 µL/mg tissue) consisting of 7 M urea, 2 M thiourea, 4% (*w*/*v*) CHAPS, 10 mM DTT, 1 mM EDTA, 1 mM PMSF, 0.2 mM Na_2_VO_3_ and 1 mM NaF. After sonication in an ice bath, the suspension was centrifuged at 12,000× *g* for 40 min at 4 °C. The protein concentration of the samples was determined by using the Bradford method [53].

### 4.3. Two-Dimensional Gel Electrophoresis (2-DE)

Five hundred micrograms of protein from hippocampal samples (TLE and control) were used for 2-DE. Linear (17 cm) pH 3–10 IPG strips (BioRad Laboratories, Hercules, CA, USA) were used for the first-dimension electrophoresis. The active rehydration was carried out for 12 h at 50 V. Isoeletric focusing (IEF) was performed using a Protean IEF cell (BioRad Laboratories, Hercules, CA, USA). After IEF, the strips were equilibrated in a buffer consisting of 50 mM Tris-HCl (pH 8.8), 6 M urea, 34% glycerol, 2% SDS, 1% DTT and 0.001% bromophenol blue. After 15 min, the stirring strips were equilibrated in a second buffer which did not contain DTT and had an additional 2.5% iodoacetamide relative to the first buffer. The equilibrated strips were then placed onto second dimension gels (12% SDS-PAGE). SDS-PAGE was performed using a Protean II xi Cell (BioRad Laboratories, Hercules, CA, USA) with a standard Tris-Glycine-SDS buffer, with a current setting of 20 mA/gel for 1 h, followed by 60 mA/gel until the bromophenol blue dye reached the end of the gel. The gels were stained using the Coomassie blue method [54]. The electrophoretic runs were made in duplicates.

### 4.4. Image Analysis for Proteome Determination

Stained gels were scanned by a GS-800 calibrated densitometer (BioRad Laboratories, Hercules, CA, USA), normalized to the background, and analyzed by PDQuest 2D-gel software (Version 8.0.1, BioRad Laboratories, Hercules, CA, USA). From selected gels, spots of interest were cut from the gel for mass spectrometry identification. For greater reliability, the identification was made in duplicates.

### 4.5. In-Gel Digestion

Excised protein spots were subjected to in-gel trypsin digestion. The spots were briefly washed by adding and removing a solution containing 100 mM (NH_4_)_2_CO_3_ with 50% acetonitrile until total discoloration. The gel fragments were dehydrated with 50 μL of pure acetonitrile in a vacuum centrifuge. Once fully dried, the gel fragments were rehydrated in 5 μL of digestion buffer consisting of 50 mM ammonium (NH_4_)_2_CO_3_ (pH 8.0) and 0.5 mg of trypsin, (Sigma-Aldrich, SP, Brazil) for 20 min at room temperature. A volume of 100 μL of 50 mM (NH_4_)_2_CO_3_ was added to all tubes and the samples were incubated overnight at 37 °C. The reaction was stopped by adding 50 μL of 0.1% trifluoroacetic acid (TFA). The samples were dehydrated in a vacuum centrifuge, re-suspended in 0.1% TFA, and analyzed by LC-ESI-MS/MS.

### 4.6. Nano-LC-ESI-MS/MS Analysis

An aliquot (4.5 μL) of digested proteins was injected into analytic columns C18 (1.7 μm), BEH 130 (100 μm × 100 mm), RP-UPLC (nanoAcquity UPLC, Waters), coupled with nano-electrospray tandem mass spectrometry on a Q-Tof Ultima API mass spectrometer (MicroMass/Waters), at a flow rate of 600 nL/min. A Symmetry C18 (180 μm × 20 mm) trapping column was used for sample desalting at a flow rate of 5 µL/min for 2 min. The gradient was 0–50% acetonitrile in 0.1% formic acid over 45 min. The instrument was operated with an MS positive mode data continuum acquisition from m/z 100–2KDa at a scan rate of 1 s and an interscan delay of 0.1 s.

Database searches for peptide identification from LC MS-MS experiments were completed with a Mascot Distiller v.2.3.2.0, 2009 (Matrix Science, Boston, MA, USA) using carbamidomethyl–cys as fixed modification (monoisotopic mass 57.0215Da). Lysine and/or arginine methylation, lysine acetylation, methionine and/or tryptophan oxidation were used as variable modification (monoisotopic mass 15.9949) and 0.1 Da MS and MSMS was applied as fragment tolerances. For protein identification, the *Homo sapiens* protein database was used.

### 4.7. Interactome

An interactome was generated using the GENEmania system (http://www.genemania.org) to identify biological functions and the respective genes that are likely involved with these functions in the network of proteins of interest. This enrichment was made based on gene ontology (GO) term annotations against the background of all genes in the organisms (*Homo sapiens*) with any gene ontology annotation [55]. A Benjamin–Hochberg FDR test using multiple corrections was used and an FDR (or “*q*-value”) was reported for each associated term. Only GO terms with a FDR greater than 0.05, at least 10 annotations in the organism, and no more than 300 to control the size of the multiple testing corrections were considered.

### 4.8. Western Blot

Western blot analysis of HSP70, H^+^-ATPase and glutathione S-transferase P were used to validate the data obtained by proteomics. Hippocampal samples of patients and control groups (N = 5/group) were homogenized using a lysis buffer containing 50 mM Tris-HCl (pH8.0), 150 mM NaCl, 0.1% SDS, 1% Triton X-100, and a 1% cocktail of protease inhibitors (Sigma). The suspension was centrifuged at 12,000× *g* for 40 min at 4 °C. Protein concentration in the samples was determined by Bradford method (Bradford, 1976). Samples of 40 µg of protein boiled in Laemmli buffer (10 min) were loaded onto a 12% acrylamide gel, separated electrophoretically and transferred to polyvinyldifluoridine (PVDF) membranes (Millipore). Membranes were incubated for 1 h in Tris buffered saline (pH 7.5) + 0.1% Tween (TBST) containing 5% skimmed milk, overnight at 4 °C with the following antibodies diluted in the same solution: mouse monoclonal anti-heat shock protein 70 (HSP70, 1:3000, Sigma) and ß-actin (1:3000, Sigma) and rabbit polyclonal anti-ATP6V1B2 (1:1000, Sigma) and anti-glutathione S-transferase P1-1 (1:1000, Calbiochem). After washing in TBST, the incubation was performed with the secondary antibody for 2 h at room temperature, a peroxidase-conjugated goat anti-rabbit (1:2000) or an anti-mouse antibody (1:5000, Chemicon, CA, USA). After three washes, chemoluminescence detection reagents (Pierce Protein Research Products, Thermo Scientific, Waltham, MA, USA) enabled visualization of peroxidase reaction products. The Densirag software was used to quantify HSP70, ATP6V1B2 and glutathione S-transferase bands (Biocom, France).

### 4.9. Statistics

Data obtained from 2-DE quantification and immunoblotting are presented as means ± standard deviation (SD). The PDQuest software analyzed the statistical differences of optical density in the proteomic study. Western blot data were analyzed by unpaired the Student´s *t*-test using GraphPad Instat Program. Statistical significance was defined at *p* < 0.05. 

## 5. Conclusions

Our results provide evidence supporting a direct link between synaptic plasticity, metabolic disturbance, oxidative stress with mitochondrial damage, disruption of the blood–brain barrier, and changes in CNS structural proteins. In addition, proteins related to cell survival were up-regulated, suggesting the presence of compensatory mechanisms in response to the activation of cascades of cell death in the pathophysiology of MTLE. DPYL2 was up-regulated in all hippocampal samples of patients with MTLE. This cytosolic protein participates in several processes of synaptic plasticity associated with MTLE, and it can be involved in axonal outgrowth as part of mechanism that predispose to seizure generation. Candidate biomarker was identified, such as GSTP1 for drug-resistance related to MTLE. GSTP1 and PARK7 were found only in MTLE patients and were absent in the control group, and GSTP1 was involved with drug-resistance. Further investigations on the role of these and other proteins identified in the present study are necessary for a more robust understanding of their roles in epileptogenesis and in the development of comorbidities. In future studies, the usage of experimental models will be helpful as they allow the study of epileptogenesis phenomena under the conditions of blockage or over-expression in the proteins of interest.

## Figures and Tables

**Table 1:** Proteins differentially expressed in the hippocampus of patients with MTLE.

IP	Protein Name	MW	Changes
5.92	Isoform 1 of Serum albumin—ALB	71317	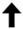
5.56	Heat shock-related 70 kDa protein 2—HSP70	70263	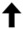
8.2	Dihydropyrimidinase-related protein 2—DPYSL2	77912	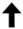
9.79	Isoform 1 of Myelin basic protein—MBP	33097	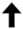
5.21	Isoform 3 of Spectrin alpha chain, brain—SPTAN1	282906	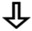
5.35	V-type proton ATPase catalytic subunit A—ATP6V1A	68660	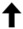
5.43	Glutathione S-transferase P—GSTP1	23569	+
6.33	Protein DJ-1—PARK7	20050	+
7.96	Dihydrolipoamide S-acethyltransferase component of pyruvate dehydrogenase complex, mitochondrial—DLAT	69466	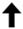

**Table 2:** Cellular and molecular functions of proteins identified in MTLE using proteomic analysis.

Proteins	Functions	References
Isoform 1 of Serum albumin—ALB	Regulation of colloidal osmotic pressure of the blood. In the brain is indicative of transient alteration of BBB and cell death.	[18,19,20]
Heat shock-related 70 kDa protein 2-HSP70	Chaperones; compensatory mechanism to neurodegeneration.	[21]
Dihydropyrimidinase- related protein 2-DPYSL2	Phosphoprotein involved with process of axonal outgrowth and regeneration of adult neurons. Its regulating the dynamics of microtubules.	[22,23]
Isoform 1 of Myelin basic protein-MBP	The presence in the brain is associated with the changes in the mechanisms of myelination and change in the permeability of the blood brain barrier.	[18,19]
Isoform 3 of Spectrin alpha chain, brain-SPTAN1	Responsible for the anchoring the NMDA receptor to the cell membrane.	[24]
V-type proton ATPase catalytic subunit A -ATP6V1A	Release of neurotransmitters and acidification of synaptic vesicles after exocytosis for recycling.	[25]
Glutathione S- transferase P-GSTP1	Antioxidant mechanisms; Inactivation of antiepileptic drugs in the liver Related to drug resistance often present in TLE.	[26,27]
Protein DJ-1-PARK7	Neuroprotection against oxidative stress.	[28,29]
Dihydrolipoamide S-acethyltransferase component of pyruvate dehydrogenase complex, mitochondrial—DLAT	Catalyzes the overall conversion of pyruvate to acetyl-CoA and CO_2_; links the glycolytic pathway to the tricarboxylic cycle.	[30]

**Table 3:** Network-wide functions including proteins of interest and interacting proteins.

Function	FDR	Genes in Network	Genes in Genome
Regulation of acyl-coa biosynthetic process	2.61 × 10^−8^	5	12
Acetyl-coa biosynthetic process from pyruvate	2.61 × 10^−8^	5	12
Regulation of cofactor metabolic process	2.61 × 10^−8^	5	13
Regulation of acetyl-coa biosynthetic process from pyruvate	2.61 × 10^−8^	5	12
Regulation of coenzyme metabolic process	2.61 × 10^−8^	5	13
Acetyl-Coa biosynthetic process	3.38 × 10^−8^	5	14
Bile acid and bile salt transport	6.3 × 10^−8^	5	16
Acetyl-Coa metabolic process	1.47 × 10^−7^	5	19
Regulation of fatty acid metabolic process	1.89 × 10^−7^	6	49
Pyruvate metabolic process	6.59 × 10^−7^	5	26
Thioester biosynthetic process	1.41 × 10^−7^	5	31
Acyl-Coa biosynthetic process	1.41 × 10^−6^	5	31
Sodium-independent organic anion transport	2.99 × 10^−6^	4	12
Bile acid metabolic process	3.08 × 10^−6^	5	37
Acyl-Coa metabolic process	1.92 × 10^−5^	5	54
Thioester metabolic process	1.92 × 10^−5^	5	54
Coenzyme biosynthetic process	3.38 × 10^−5^	5	61
Regulation of cellular ketone metabolic process	3.54 × 10^−5^	6	129
Mitochondrial matrix	8.39 × 10^−5^	7	257
Monocarboxylic acid transport	1.00 × 10^−4^	5	78
Regulation of lipid metabolic process	1.18 × 10^−4^	6	162
Cofactor biosynthetic process	1.24 × 10^−4^	5	83
Cellular ketone metabolic process	1.24 × 10^−4^	6	166
Steroid metabolic process	3.18 × 10^−4^	6	196
Fatty acid metabolic process	4.45 × 10^−4^	6	209
Coenzyme metabolic process	1.11 × 10^−3^	5	133
Oxidoreductase complex	2.23 × 10^−3^	4	68
Carboxylic acid transport	3.06 × 10^−3^	5	166
Organic acid transport	3.13 × 10^−3^	5	168
Cofactor metabolic process	3.90 × 10^−3^	5	177
Hydrogen ion transmembrane transporter activity	5.86 × 10^−3^	3	28
Interaction with host	5.86 × 10^−3^	4	90
Ferric iron transport	7.86 × 10^−3^	3	32
Transferrin transport	7.86 × 10^−3^	3	32
Proton-transporting two-sector atpase complex	7.86 × 10^−3^	3	32
Trivalent inorganic cation transport	7.86 × 10^−3^	3	32
Blood microparticle	1.03 × 10^−2^	4	108
Iron ion transport	1.59 × 10^−2^	3	41
Organic anion transport	1.72 × 10^−2^	5	254
Phagosome maturation	1.74 × 10^−2^	3	43
Cellular respiration	2.57 × 10^−2^	4	140
Negative regulation of extrinsic apoptotic signaling pathway	3.12 × 10^−2^	3	53
Cellular iron ion homeostasis	3.99 × 10^−2^	3	58
Transition metal ion transport	4.88 × 10^−2^	3	63
Iron ion homeostasis	4.88 × 10^−2^	3	63
Cellular transition metal ion homeostasis	7.85 × 10^−2^	3	75
Proton transport	7.85 × 10^−2^	3	75
Hydrogen transport	8.31 × 10^−2^	3	77
Regulation of cellular carbohydrate metabolic process	9.45 × 10^−2^	3	81

The FDR (false discovery rate) report the significance level. The data show the number of genes in the network and the total number of genes (from genome) involved with the function.

**Table 4:** Clinical and demographic data of patients with MTLE subjected to hippocampectomy.

Data	
Number of patients	6
Age at surgery (Mean ± SD)	42.2 ± 9.9
Gender-females	2
Age at epilepsy onset-months (mean ± SD)	14.7 ± 8.3
Years of epilepsy at surgery (mean ± SD)	18 ± 10
Family history of epilepsy (%)	33.3
Presence of febrile seizures (%)	50
Diagnosis of psychiatric disorders	3

SD: Standard deviation.

**Table 5:** Clinical and demographic data of autopsied patients (control samples).

Data	
Number of patients	10
Age at autopsy (Mean ± SD)	56 ± 18
Gender-females	4
Postmortem period	<6 h
Changes in central nervous system	No
Family history of epilepsy (%)	No
Diagnosis of psychiatric disorders	No

SD: Standard deviation.

## References

[1] (2018). WHO. http://www.who.int/news-room/fact-sheets/detail/epilepsy.

[2] Engel J. (2001). A Proposed Diagnostic Scheme for People with Epileptic Seizures and with Epilepsy: Report of the ILAE Task Force on Classification and Terminology. Epilepsia.

[3] Mathern G.W., Pretorius J.K., Babb T.L. (1995). Influence of the type of initial precipitating injury and at what age it occurs on course and outcome in patients with temporal lobe seizures. J. Neurosurg..

[4] Whelan C.D., Altmann A., Botia J.A., Jahanshad N. (2018). Structural brain abnormalities in the common epilepsies assessed in a worldwide ENIGMA study. Brain.

[5] Graebenitz S.P., Cerina M., Lesting J.R., Kedo O., Gorji A., Pannek H., Hans V., Zilles K., Pape H.C., Speckmann E.J. (2017). Directional spread of activity in synaptic networks of the human lateral amygdala. Neuroscience.

[6] Chen S.D., Wang Y.L., Liang S.F., Shaw F.Z. (2017). Rapid amygdala kindling causes motor seizure and comorbidity of anxiety- and depression-like behaviors in rats. Front. Neurol..

[7] French J.A. (2017). Refractory epilepsy: Clinical overview. Epilepsia.

[8] Wotton C.J., Goldacre M.J. (2012). Coexistence of schizophrenia and epilepsy: Record-linkage studies. Epilepsia.

[9] Ravizza T., Onat F.Y., Brooks-Kayal A.R., Depaulis A., Galanopoulou A.S., Mazarati A., Hans V., Zilles K., Pape H., Speckmann E. (2017). WONOEP appraisal: Biomarkers of epilepsy-associated comorbidities. Epilepsia.

[10] Czech T., Yang J.W., Csaszar E., Kappler J., Baumgartner C., Lubec G. (2004). Reduction of Hippocampal Collapsin Response Mediated Protein-2 in Patients with Mesial Temporal Lobe Epilepsy. Neurochem. Res..

[11] Eun J.P., Choi H.Y., Kwak Y.G. (2004). Proteomic analysis of human cerebral cortex in epileptic patients. Exp. Mol. Med..

[12] Xiao F., Chen D., Lu Y., Xiao Z., Guan L., Yuan J., Wang L., Xi Z., Wang X. (2009). Proteomic analysis of cerebrospinal fluid from patients with idiopathic temporal lobe epilepsy. Brain Res..

[13] Yang J.W., Czech T., Yamada J., Csaszar E., Baumgartner C., Slavc I., Lubec G. (2004). Aberrant cytosolic acyl-CoA thioester hydrolase in hippocampus of patients with mesial temporal lobe epilepsy. Amino Acids.

[14] Yang J.W., Czech T., Gelpi E., Lubec G. (2005). Extravasation of plasma proteins can confound interpretation of proteomic studies of brain: A lesson from apo A-I in mesial temporal lobe epilepsy. Mol. Brain Res..

[15] He S., Wang Q., He J., Pu H., Yang W., Ji J. (2006). Proteomic analysis and comparison of the biopsy and autopsy specimen of human brain temporal lobe. Proteomics.

[16] Mériaux C., Franck J., Park D.B., Quanico J., Kim Y.H., Chung C.K., Park Y.M., Steinbusch H., Salzet M., Fournier I. (2014). Human temporal lobe epilepsy analyses by tissue proteomics. Hippocampus.

[17] Persike D.S., Lima M.L., Amorim R.P., Cavalheiro E.A., Yacubian E.M.T., Centeno R.S., Carrete H., Schenkman S., Canzian M., Fernandes M.J.S. (2012). Hippocampal Proteomic Profile in Temporal Lobe Epilepsy. J. Epilepsy Clin. Neurophysiol..

[18] Huang Z., Zhou Y., Xiao B., Wu J., Wu X., Yang P., Wu L. (2008). Proteomic screening of postsynaptic density proteins related with temporal lobe epilepsy. Chin. Med. J..

[19] Marchi N., Teng Q., Ghosh C., Fan Q., Nguyen M.T., Desai N.K., Bawa H., Rasmussen P., Masaryk T.K., Janigro D. (2010). Blood–brain barrier damage, but not parenchymal white blood cells, is a hallmark of seizure activity. Brain Res..

[20] Liu Z., Liu J., Wang S., Liu S., Zhao Y. (2016). Neuronal uptake of serum albumin is associated with neuron damage during the development of epilepsy. Exper. Ther. Med..

[21] Mayer M.P., Bukau B. (2005). Hsp70 chaperones: Cellular functions and molecular mechanism. Cell. Mol. Life Sci..

[22] Castegna A., Aksenov M., Thongboonkerd V., Klein J.B., Pierce W.M., Booze R., Markesbery W.R., Butterfield D.A. (2002). Proteomic identification of oxidatively modified proteins in Alzheimer’s disease brain. Part II: Dihydropyrimidinase-related protein 2, alpha-enolase and heat shock cognate 71. J. Neurochem..

[23] Gu Y., Ihara Y. (2000). Evidence that collapsin response mediator protein-2 is involved in the dynamics of microtubules. J. Biol. Chem..

[24] Wechsler A., Teichberg V.I. (1998). Brain spectrin binding to the NMDA receptor is regulated by phosphorylation, calcium and calmodulin. EMBO J..

[25] Toei M., Saum R., Forgac M. (2010). Regulation and isoform function of the V-ATPases. Biochemistry.

[26] Sharma R., Yang Y., Sharma A., Awasthi S., Awasthi Y.C. (2004). Antioxidant Role of Glutathione S-Transferases: Protection Against Oxidant Toxicity and Regulation of Stress-Mediated Apoptosis. Antioxid. Redox Signal..

[27] Shang W., Liu W.H., Zhao X.H., Sun Q.J., Bi J.Z., Chi Z.F. (2008). Expressions of glutathione S-transferase alpha, mu, and pi in brains of medically intractable epileptic patients. BMC Neurosci..

[28] Taira T., Saito Y., Niki T., Iguchi-Ariga S.M., Takahashi K., Ariga H. (2004). DJ-1 has a role in antioxidative stress to prevent cell death. EMBO Rep..

[29] Junn E., Jang W.H., Zhao X., Jeong B.S. (2009). Mouradian MM. Mitochondrial localization of DJ-1 leads to enhanced neuroprotection. J. Neurosci. Res..

[30] Berg J.M., Tymoczko J.L., Stryer L. (2002). Biochemistry.

[31] Surguchov A., Surgucheva I., Sharma M., Sharma R., Singh V. (2017). Pore-forming proteins as mediators of novel epigenetic mechanism of epilepsy. Front. Neurol..

[32] Marques-Carneiro J.E., Persike D.S., Litzahn J.J., Fernandes M.J.S. (2017). Hippocampal Proteome of rats subjected to the Li-pilocarpine epilepsy model and effect of carisbamate treatment. Pharmaceuticals.

[33] Morimura R., Nozawa K., Tanaka H., Ohshima T. (2013). Phosphorylation of Dpsyl2 (CRMP2) and Dpsyl3 (CRMP4) is required for positioning of caudal primary motor neurons in the zebrafish spinal cord. Dev. Neurobiol..

[34] Ujike H., Sakai A., Nakata K., Tanaka Y., Kodaka T., Okahisa Y., Harano M., Inada T., Yamada M., Komiyama T. (2006). Association study of the dihydropyrimidinase-related protein 2 gene and methamphetamine psychosis. Ann. N. Y. Acad. Sci..

[35] Curia G., Lucchi C., Vinet J., Gualtieri F., Marinelli C., Torsello A., Costantino L., Biagini G. (2014). Pathophysiogenesis of mesial temporal lobe epilepsy: Is prevention of damage antiepileptogenic?. Curr. Med. Chem..

[36] Gray W.P., Sundstrom L.E. (1998). Kainic acid increases the proliferation of granule cell progenitors in the dentate gyrus of the adult rat. Brain Res..

[37] Buga A.M., Vintilescu R., Balseanu A.T., Pop O.T., Streba C. (2012). Repeated PTZ treatment at 25-day intervals leads to a highly efficient accumulation of doublecortin in the dorsal hippocampus of rats. PLoS ONE.

[38] Schmoll H., Badan I., Grecksch G., Walker L., Kessler C., Popa-Wagner A. (2003). Kindling status in sprague-dawley rats induced by pentylenetetrazole. Am. J. Pathol..

[39] Zhong Q., Ren B.X., Tang F.H. (2016). Neurogenesis in the hippocampus of patients with temporal lobe epilepsy. Curr. Neurol. Neurosci..

[40] Silver J.T., Noble E.G. (2012). Regulation of survival gene hsp70. Cell Stress Chaperon..

[41] Planas A.M., Soriano M.A., Ferrer I., Rodríguez F.E. (1995). Kainic acid-induced heat shock protein-70, mRNA and protein expression is inhibited by MK-801 in certain rat brain regions. Eur. J. Neurosci..

[42] Wilkens S., Zhang Z., Zheng Y. (2005). A structural model of the vacuolar ATPase from transmission electron microscopy. Micron.

[43] Wang Y., Floor E. (1998). Hydrogen peroxide inhibits the vacuolar H+-ATPase in brain synaptic vesicles at micromolar concentrations. J. Neurochem..

[44] Li Z., Burrone J., Tyler W.J., Hartman K.N., Albeanu D.F., Murthy V.N. (2005). Synaptic vesicle recycling studied in transgenic mice expressing synaptopHluorin. Proc. Natl. Acad. Sci. USA.

[45] Chan J.Y., Chan S.H. (2015). Activation of endogenous antioxidants as a common therapeutic strategy against cancer, neurodegeneration and cardiovascular diseases: A lesson learnt from DJ-1. Pharmacol. Ther..

[46] Zhou W., Barkow J.C., Freed C.R. (2017). Running wheel exercise reduces α-synuclein aggregation and improves motor and cognitive function in a transgenic mouse model of Parkinson’s disease. PLoS ONE.

[47] Kovac S., Kostova A.T.D., Herrmann A.M., Melzer N., Meuth S.G., Gorji A. (2017). Metabolic and homeostatic changes in seizures and acquired epilepsy-mitochondria, calcium dynamics and reactive oxygen species. Int. J. Mol. Sci..

[48] Seyfried B.T., Mantis J.G., Todorova M.T., Greene A.E., Schwartzkroin P.A. (2009). Dietary management of epilepsy: Role of glucose and ketone bodies. The Encyclopedia of Basic Epilepsy Research.

[49] Giordano C., Marchio M., Timofeeva E., Biagini G. (2014). Neuroprotective peptides as putative mediators of antiepileptic ketogenic diets. Front. Neurol..

[50] You Y., Bai H., Wang C., Chen L.W., Liu B., Zhang H., Gao G.D. (2011). Myelin damage of hippocampus and cerebral cortex in rat pentylenetetrazol model. Brain Res..

[51] Keck M., Dijk R.M., Deeg C.A., Kistler K., Walker A., Rüden E.L., Russman V., Hauck S.M., Potschka H. (2018). Proteomic profiling of epileptogenesis in a rat model: Focus on cell stress, extracellular matrix and angiogenesis. Neurobiol. Dis..

[52] Vinet J., Costa A.M., Salinas-Navarro M., Leo G., Moons L., Arckens L., Biagini G. (2018). A Hydroxypyrone-Based Inhibitor of Metalloproteinase-12 Displays Neuroprotective Properties in Both *Status Epilepticus* and Optic Nerve Crush Animal Models. Int. J. Mol. Sci..

[53] Bradford M.M. (1976). A rapid and sensitive method for the quantitation of microgram quantities of protein utilizing the principle of protein-dye binding. Anal. Biochem..

[54] Candiano G., Bruschi M., Musante L., Santucci L., Ghiggeri G.M., Carnemolla B., Orecchia P., Zardi L., Righetti P.G. (2004). Blue silver: A very sensitive colloidal Coomassie G-250 staining for proteome analysis. Electrophoresis.

[55] Carnielli C.M., Winck F.V., Leme A.F.P. (2015). Functional annotation and biological interpretation of proteomics data. BBA-Proteins Proteom..

